# Case Report: Successful late-line pralsetinib treatment in an ALK-rearranged lung adenocarcinoma patient with KIF5B-RET fusion resistant to alectinib

**DOI:** 10.3389/fgene.2025.1569912

**Published:** 2025-06-17

**Authors:** Feng Jin, Chenyang Wang, Fang Yang, Shubin Wang, Fen Wang

**Affiliations:** ^1^ Department of Medical Oncology, Peking University Shenzhen Hospital, Shenzhen, China; ^2^ Shenzhen University, Shenzhen, China

**Keywords:** lung adenocarcinoma, *KIF5B-RET*, alectinib, pralsetinib, case report, ALK-rearranged

## Abstract

Anaplastic lymphoma kinase (*ALK*) fusion, an oncogenic driver alteration, accounts for 5%–6% of non-small cell lung cancer (NSCLC) patients. *ALK* tyrosine kinase inhibitors (TKIs) provide significant clinical benefit in advanced ALK-rearranged NSCLC. However, acquired resistance to *ALK* TKIs inevitably arises, and the underlying mechanisms remain incompletely elucidated. This report describes a stage IV lung adenocarcinoma (LUAD) patient with *ALK*-rearranged who developed *KIF5B-RET* fusion-mediated resistance following second-line alectinib therapy. The patient achieved a partial response (PR) to third-line pralsetinib, sustained for 4 months. This case highlights *KIF5B-RET* fusion as a potential resistance mechanism post alectinib treatment and suggested = pralsetinib, a *RET* inhibitor, as a viable therapeutic option in this context. These findings contribute to the evolving understanding of resistance management strategies in *ALK*-rearranged NSCLC.

## Introduction

Non-small cell lung cancer (NSCLC) accounts for 80%–85% of lung cancer cases and remains a leading cause of cancer-related mortality worldwide (Siegel et al., 2022). Advances in next-generation sequencing (NGS) technology and precision oncology have revolutionized therapeutic strategies, enabling targeted therapies to become a cornerstone of NSCLC management (Tan and Tan, 2022). Among oncogenic driver alterations, *anaplastic lymphoma kinase (ALK)* fusions are identified in 5%–6% of NSCLC patients and serve as critical therapeutic targets (Yang et al., 2023). Multiple *ALK* tyrosine kinase inhibitors (TKIs) with excellent efficacy, including first-generation crizotinib, second-generation ceritinib, alectinib, and brigatinib, and third-generation lorlatinib, have been approved for treating ALK-rearranged NSCLC patients. Despite their clinical success, the long-term efficacy of these agents is frequently hindered by the inevitable development of resistance, the mechanisms of which remain incompletely characterized. This case report describes a stage IV *ALK*-rearranged lung adenocarcinoma (LUAD) patient who developed *KIF5B-RET* fusion-mediated resistance to second-line alectinib therapy. Notably, the patient achieved a partial response third-line pralsetinib, a selective *RET* inhibitor, with a duration of 4 months, provide novel insights into resistance mechanisms and salvage therapeutic options.

## Case description

In September 2020, a 50-year-old male presented to his local hospital with persistent cough. Initial chest computed tomography (CT) imagine identified a nodule in the dorsal segment of the left lower lung lobe. Subsequent followed-up scans performed due to progressive symptoms revealed a 11 mm × 10 mm lesion in the left lower lung and enlarged mediastinal lymph nodes. A lymph node biopsy at this stage confirmed poorly differentiated LUAD with genetic testing demonstrating wild-type *EGFR* exons (18-21) and *ALK*. Notably, the local hospital omitted tumor marker assessment (e.g., carcinoembryonic antigen) and immunohistochemical analysis of biopsy specimens.

**FIGURE 1:**
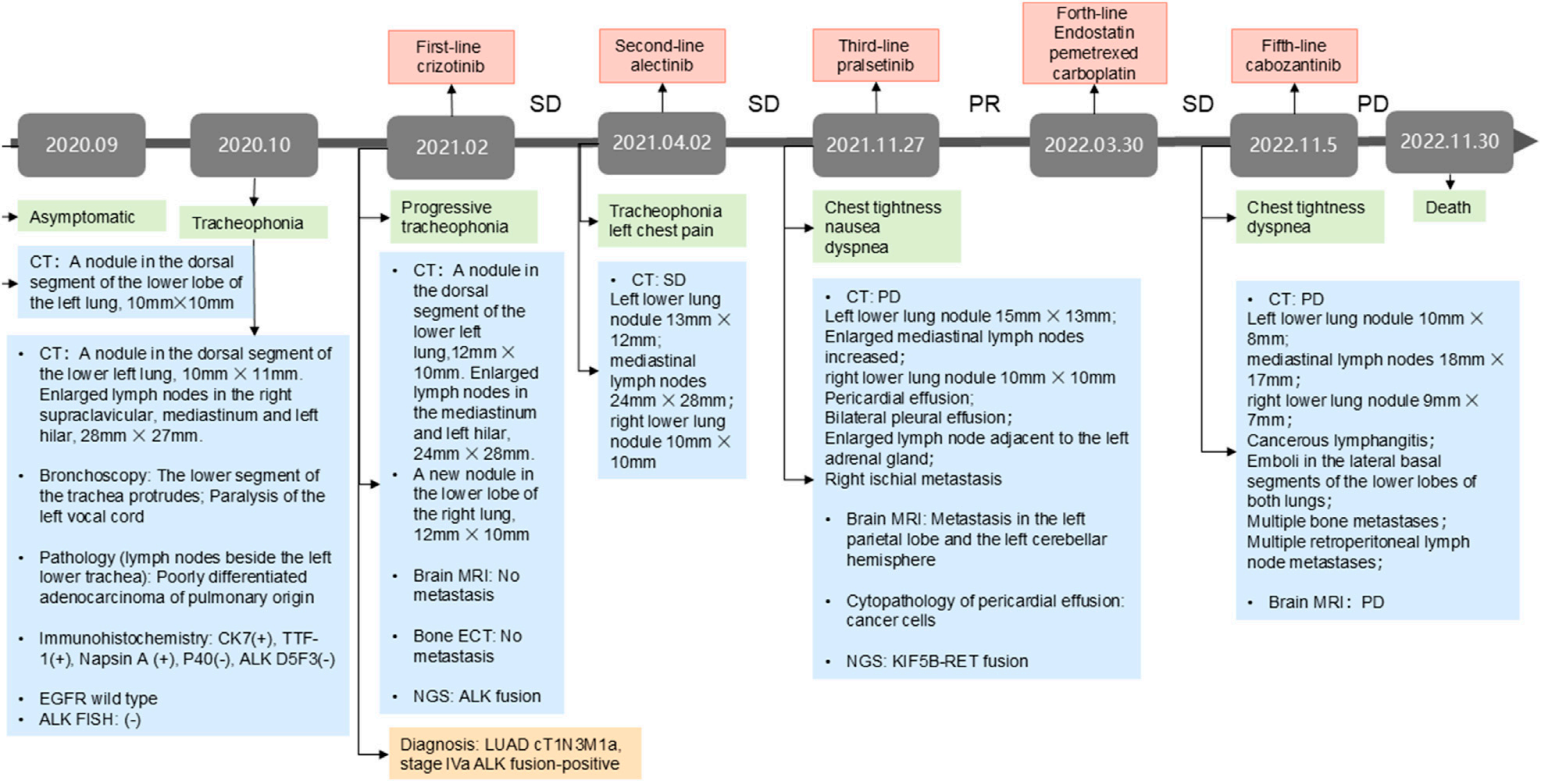
The treatment milestone of the patient. NGS, next-generation sequencing; PR, partial response; SD, stable disease; PD, progressive disease; TMB, tumor mutational burden; LUAD, lung adenocarcinoma. CT, computed tomography; MRI: magnetic resonance imaging.

In February 2021, the patient was referred to our institution with worsening tracheophonia. Repeat chest CT scans showed a left lower lung lesion, enlarged right supraclavicular and mediastinal lymph nodes, and a new in the right lower lung nodule. Magnetic resonance imaging (MRI) and bone emission computed tomography scan revealed no evidence of brain or bone metastases. NGS (11- gene panel covering 11 oncogenic driver genes recommended by the NCCN guidelines) of the biopsy tissue from the 4L lymph node biopsy tissue identified an *echinoderm microtubule-associated protein-like 4 (EML4)-ALK*- rearranged (allele frequency: 16%). The patient was diagnosed with stage IVa (cT1N3M1a) *ALK* rearranged LUAD. The treatment timeline is summarized in Figure 1.

Owing to financial constraints, the patient received first-line crizotinib (250 mg twice daily) from February to March 2021. While imaging indicated stable disease, his clinical symptoms, including tracheophonia and left chest pain, progressively deteriorated. Second-line therapy with alectinib (600 mg twice daily) was initiated, achieving stable disease until November 2021, when the patient developed chest tightness, nausea, and dyspnea. Chest CT at progression demonstrated significant mediastinal lymphadenopathy, pericardial effusion and bilateral pleural effusions. Brain MRI confirmed new metastasis lesion. Concurrently, the primary left lower lung lesion and right lung metastatic nodule exhibited slight enlargement. Laboratory findings revealed severe transaminitis (ALT and AST >1000 U/L) and jaundice.

Alectinib was discontinued due to hepatotoxicity, and the patient received glutathione and polyene phosphatidylcholine for transaminase reduction. Pericardiocentesis yielded 200–300 mL of hemorrhagic pericardial effusion daily, with cytopathology confirmed malignant cell. NGS of the pericardial effusion (520-gene panel, Burning Rock Biotech, Guangzhou, China)detected the original *ALK* rearrangement, and a novel *KIF5B-RET* fusion (K15:R12 allele frequency AF: 31.86%, tumor mutation burden:4.99 mutations/Mb).

**FIGURE 2:**
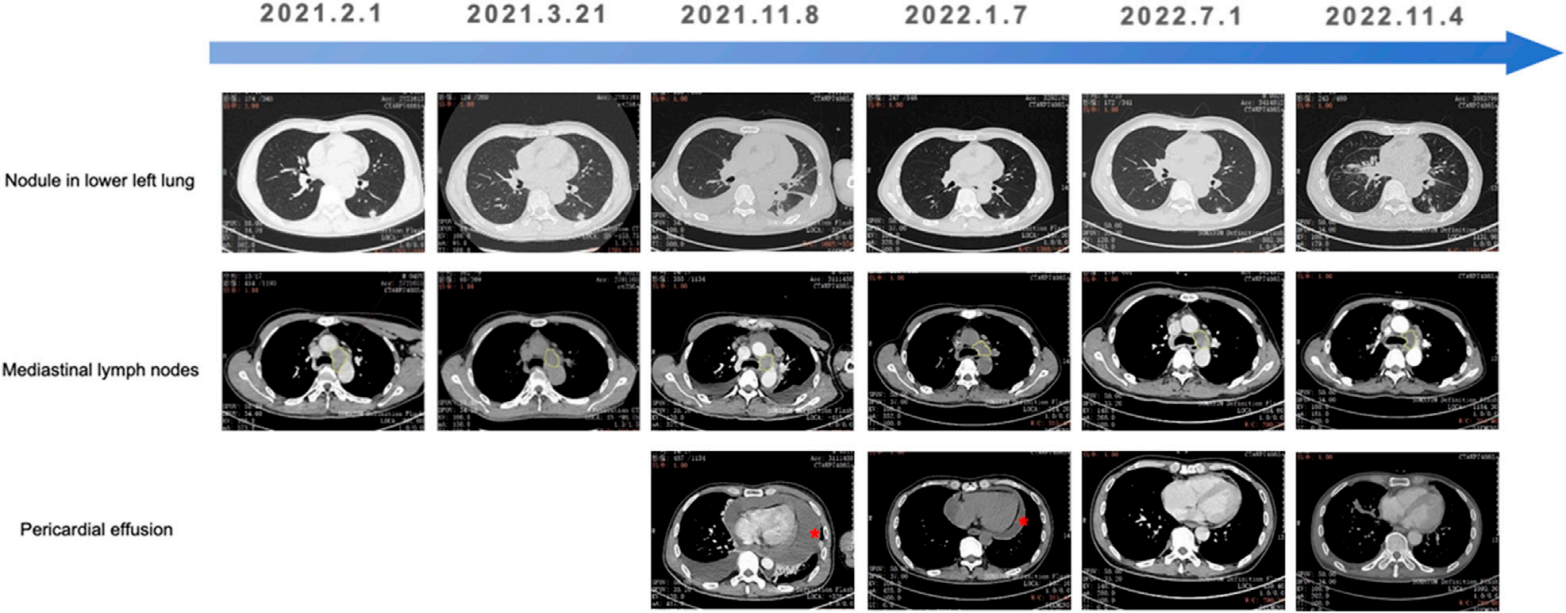
Longitudinal CT imaging of the patient’s thoracic lesions.

Given pralsetinib’s approval for *RET*-fusion-positive NSCLC, third-line pralsetinib (400 mg daily) was administered from November 2021, to March 2022, achieving partial response (RECIST 1.1). Treatment was discontinued due to financial constraints, and subsequent therapy include Endostar (recombinant human endostatin injection) combined with pemetrexed-carboplatin chemotherapy. During chemotherapy, the patient required hospitalization for zoledronic acid (bone metastasis management) and intrathoracic cisplatin (pleural effusion control). Disease progression (PD) occurred after five chemotherapy cycles. Cabozantinib (140 mg daily) was initiated on November 2022, but was discontinue due to severe adverse reactions. Palliative care was initiated following confirmed progression on chest CT, and the patient succumbed to the disease in late November 2022. Longitudinal CT imaging of the patient’s thoracic lesions are shown in Figure 2.

## Discussion

To our knowledge, this represents the first documented case of *KIF5B-RET* fusion as a resistance mechanism to alectinib in *ALK*-rearranged NSCLC. Alectinib, a second-generation *ALK* TKI has been widely used in patients progressing on crizotinib, supported by its robust efficacy reported in the phase III clinical trial ALUR study (Novello et al., 2018). In this case, the patient derived a progression-free survival (PFS) of 7 months from alectinib after failing first-line crizotinib.

While first- and next-generation *ALK* inhibitors prolong survival in ALK rearrangements NSCLC, resistance remains inevitably (Okada et al., 2019). Prior studies indicate that over 50% of alectinib-resistant tumors harbor secondary *ALK* mutations, with *ALK G1202R* (29% prevalence), *ALK I1171T/N/S*, *ALK V1180L*, and *ALK L1196M* (Gainor et al., 2016; Haratake et al., 2021). Additionally, *MET* overexpression or amplification contributes to acquired resistance alectinib through bypass signaling activation (Haratake et al., 2021). In NSCLC after multi-line targeted therapy, there is often activation of multiple driver genes and more potential treatment targets. Therefore, NGS using a broader gene panel has potential benefits for these patients. In this case, the patient progressing on alectinib underwent NGS on pericardial effusion, which revealed the presence of *KIF5B-RET* fusion without secondary *ALK* mutation or *MET* amplification. *RET* rearrangement, observed in 1%–3% of treatment-naïve NSCLCs (Tan and Tan, 2022), have been reported in EGFR-TKIs such as osimertinib (Leonett et al., 2019; Offin et al., 2018). However, reports linking *RET* fusions to *ALK* TKI resistance are scarce. Yan et al. reported a case of NCOA4-RET fusion mutation occurring after acquired resistance to alectinib (Yan et al., 2024). Both previous studies and our case suggest that KIF5B-RET fusion could contribute to resistance to the second-generation *ALK* TKI alectinib. The patient was observed to benefit from third-line pralsetinib.

Liquid biopsy has emerged as a minimally invasive tool for molecular profiling in NSCLC, utilizing peripheral blood, pleural effusion, and cerebrospinal fluid to circumvent tumor heterogeneity (Ye et al., 2019; He et al., 2022). Multiple retrospective analyses have demonstrated that NGS performed on body fluids, including pericardial effusion, exhibits high concordance with tissue-based testing in detecting driver gene alterations (Wei et al., 2016; Zhang et al., 2019). While pericardial effusion analysis in this case successfully identified *KIF5B-RET* fusion, its utility is constrained by limited availability and potential sampling bias. Future studies should validate the concordance between pericardial effusion and tissue-based genomic profiles in larger cohorts.

The next-generation sequencing (NGS) performed in this study was DNA-based, utilizing a panel covering 11 oncogenic driver genes recommended by the NCCN guidelines for lung cancer, which includes *RET* fusion detection. While the *KIF5B-RET* fusion was identified at disease progression (allele frequency: 31.86%), baseline NGS analysis of the initial lymph node biopsy (February 2021) confirmed wild-type *ALK* and *EGFR* but did not detect *RET* alterations. Notably, RNA-based NGS, which offers higher sensitivity for fusion detection, was not performed due to insufficient tissue availability.

It is important to acknowledge that discordance between DNA- and RNA-based NGS has been reported in prior studies, primarily due to differences in genomic breakpoints or low tumor content in samples (Li et al., 2020). Thus, while our findings strongly suggest *KIF5B-RET* fusion as an acquired resistance mechanism post-alectinib, the possibility of a pre-existing subclonal *RET* fusion below the detection threshold of DNA-NGS at baseline cannot be entirely excluded. Future studies incorporating paired RNA sequencing or more sensitive assays (e.g., digital PCR) in longitudinal samples would help clarify the origin of this alteration.

There are some limitations associated with this study. Firstly, it just a case report involves only one patient. More evidence is needed to investigate whether the presence of *KIF5B-RET* is one of the mechanisms underlying resistance to alectinib. Secondly, the concordance of genomic profiling between pericardial effusion and paired tissue samples derived from NSCLC patients should be investigated in a large cohort study.

The emergence of *KIF5B-RET* fusion as a resistance mechanism in this case underscores the complexity of bypass signaling in *ALK*-rearranged NSCLC. Beyond genomic alterations, epigenetic dysregulation—including DNA methylation, histone modifications, and chromatin remodeling—has been increasingly recognized as a driver of therapeutic resistance and tumor evolution (Jones and Baylin, 2007; Sharma et al., 2010). For instance, hypermethylation of tumor suppressor genes (e.g., *CDKN2A*) or hypomethylation of oncogenic promoters (e.g., *RET*) may synergize with fusion events to sustain proliferative signaling (Heller et al., 2013). Recent studies suggest that *ALK* fusions themselves can modulate the epigenetic landscape by recruiting histone acetyltransferases (HATs) or methyltransferases (e.g., EZH2), thereby promoting oncogene addiction and resistance to TKIs (Lovly and Shaw, 2014). In *RET*-rearranged tumors, aberrant DNA methylation patterns have been linked to enhanced *RET* transcription and pathway activation, independent of fusion allele frequency (Gautschi et al., 2017). These findings highlight the potential interplay between genetic and epigenetic mechanisms in mediating resistance.

Notably, *RET* fusions may exploit epigenetic machinery to amplify downstream signaling. Preclinical models demonstrate that *RET* fusion proteins recruit histone deacetylases (HDACs) to repress negative regulators of the MAPK pathway, such as *DUSP6*, fostering sustained ERK activation (Drilon et al., 2018). Similarly, *ALK* fusions have been shown to induce global DNA hypomethylation via downregulation of DNMT3A, facilitating the expression of pro-metastatic genes (Wata et al., 2015). In this patient, while RNA-based epigenetic profiling was not performed, the high allele frequency of *KIF5B-RET* (31.86%) and its temporal association with alectinib resistance suggest that epigenetic co-drivers—if present—could have amplified *RET*-dependent survival signals.

Targeting epigenetic modifiers represents a promising avenue to overcome resistance in *ALK/RET*-altered NSCLC. HDAC inhibitors (e.g., panobinostat) and DNA hypomethylating agents (e.g., azacitidine) have shown preclinical efficacy in restoring TKI sensitivity by re-sensitizing resistant clones to apoptosis (Topper et al., 2017). For example, targeting the menin-PRC2 complex (which includes EZH2) suppresses lung adenocarcinoma growth by mediating H3K27me3-dependent silencing of the oncogenic growth factor pleiotrophin (PTN) (Gao et al 2009). This highlights the therapeutic potential of disrupting PRC2-mediated epigenetic silencing in this context. In *RET*-fusion-positive cancers, BET inhibitors (e.g., JQ1) disrupt BRD4-mediated transcriptional elongation of *RET*, potentiating the effects of *RET* TKIs (Puissant et al., 2013). Future studies should explore whether adjunct epigenetic therapy could extend the durability of pralsetinib in patients with *RET*-mediated resistance.

This case highlights *KIF5B-RET* fusion as a putative resistance mechanism to alectinib, while underscoring the need to investigate epigenetic contributors to *ALK/RET* pathway dysregulation. The integration of pharmacoepigenetic approaches—such as DNA methylation profiling or HDAC inhibition—into resistance management strategies may uncover novel therapeutic vulnerabilities. We propose that longitudinal epigenetic profiling of liquid biopsy samples (e.g., cfDNA or pericardial effusion) could identify dynamic changes in chromatin modifiers or methylation patterns associated with resistance. Furthermore, clinical trials evaluating combinations of *RET/ALK* inhibitors with epigenetic agents (e.g., HDAC or EZH2 inhibitors) are warranted to determine whether such strategies can delay or reverse resistance in molecularly defined subsets.

## Data Availability

The raw data supporting the conclusions of this article will be made available by the authors, without undue reservation.
